# Cyber security threats in the microbial genomics era: implications for public health

**DOI:** 10.2807/1560-7917.ES.2020.25.6.1900574

**Published:** 2020-02-13

**Authors:** Iliya Fayans, Yair Motro, Lior Rokach, Yossi Oren, Jacob Moran-Gilad

**Affiliations:** 1 Department of Software and Information Systems Engineering, Faculty of Engineering Sciences, Ben Gurion University of the Negev, Beer Sheva, Israel; 2 Department of Health Systems Management, School of Public Health, Faculty of Health Sciences, Ben-Gurion University of the Negev, Beer-Sheva, Israel

**Keywords:** Next Generation Sequencing, cyber, Security, microbiology, threat assessment

## Abstract

Next generation sequencing (NGS) is becoming the new gold standard in public health microbiology. Like any disruptive technology, its growing popularity inevitably attracts cyber security actors, for whom the health sector is attractive because it combines mission-critical infrastructure and high-value data with cybersecurity vulnerabilities. In this Perspective, we explore cyber security aspects of microbial NGS. We discuss the motivations and objectives for such attack, its feasibility and implications, and highlight policy considerations aimed at threat mitigation. Particular focus is placed on the attack vectors, where the entire process of NGS, from sample to result, could be vulnerable, and a risk assessment based on probability and impact for representative attack vectors is presented. Cyber attacks on microbial NGS could result in loss of confidentiality (leakage of personal or institutional data), integrity (misdetection of pathogens) and availability (denial of sequencing services). NGS platforms are also at risk of being used as propagation vectors, compromising an entire system or network. Owing to the rapid evolution of microbial NGS and its applications, and in light of the dynamics of the cyber security domain, frequent risk assessments should be carried out in order to identify new threats and underpin constantly updated public health policies.

## Introduction

Next generation sequencing (NGS) is an emerging technology in the field of public health microbiology [1]. Whole genome sequencing (WGS) of pathogens has recently gained acceptance as a new gold standard in microbiology for different pathogens and scenarios; it allows the unprecedented characterisation of pathogens with respect to taxonomy, antimicrobial resistance, virulence attributes and genotyping [2]. Among many other advantages, it is expected to reduce the time from diagnosis to clinical treatment, improve surveillance and outbreak investigation and facilitate data sharing in public health [3]. The adoption of WGS is rapidly increasing thanks to a dramatic reduction in the cost of DNA sequencing [4]. The continuous development in the field of metagenomics suggests that NGS could soon be harnessed on a routine basis for culture-independent microbiology, which is expected to further improve surveillance and management of infectious diseases [5].

As with any disruptive technology, growing popularity of a technology will inevitably attract the interest of malicious actors who will try to abuse it, at individual or state level. Painfully bright examples of this recurring pattern involved major disruptions in Internet services worldwide [6] or malicious software specifically designed to steal cryptocurrency wallets in the wake of Bitcoin’s rise [7]. The collective experience in the field of cybersecurity so far suggests that for a new technology not to become an immediate hazard, security should be integrated as early as possible and periodic security audits should be carried out throughout its whole lifecycle [8]. The costs of sequencing continue to drop, allowing efforts to introduce sequencing globally, even into low resource settings. Moreover, small footprint benchtop sequencers and, even more importantly, portable sequencers are being developed [9]. These trends indicate that in the near future, increasing proportions of microbial sequence data will be generated outside of the traditional laboratory setting, such as in the field during investigation, at the bedside and even in consumer homes and other unorthodox locations (e.g. in outer space [10]).

In this Perspective, we explore cyber security aspects of microbial NGS. We discuss the motivations and objectives for a possible attack, its feasibility and implications, and highlight policy considerations aimed at mitigating this growing threat.

## Medicine and cyber security

In recent years, a sharp rise in cyber attacks on smart medical equipment had been observed [11] as part of the more general trend of increased cyber attacks on Internet-connected devices, including smart home devices such as locks, cameras, lights and speakers. Computerised medical equipment is an attractive target for malicious cyber activity, as it is among a rapidly shrinking group of industries which combine mission-critical infrastructure and high-value data (e.g. personal health records), with relatively weak cybersecurity standards [12]. In the context of medical devices, cyber threats could be targeting a specific facility or organisation, such as the recent incident that involved hospitals in the United Kingdom [13], or involve a supply chain attack targeting less secure elements in an organisational supply network [14]. An adversary might carry out a supply chain attack by first compromising a network or device-providing service [15]. Cyber security must therefore be a core part of a medical product’s lifecycle and, in particular, integrated into the product’s design from its inception and not as an afterthought. Traditionally, the responsibility for the security of medical devices lies with the device manufacturer, while the responsibility for sensitive information is in the hands of medical institutions.

The rapid growth of machine learning applications and data analytics in medicine are also of great concern with respect to cyber security, especially in the face of adversarial learning – an advanced offensive technique designed to fool models based on machine learning that is applicable to medical information technology systems [16]. Recent studies in the field of adversarial learning have demonstrated successful attacks on medical devices such as imaging technology [17]. In an era of digital transformation of healthcare, cyber threats are unavoidable and effective cyber security requires a major investment in infrastructure, personnel and governance [12].

While cyber attacks on microbial NGS have not been reported to date, a practical attack has been performed compromising a computer as a part of an NGS pipeline via a specially synthesised DNA sequence [18], which suggests that this avenue deserves more attention and that microbial NGS has unique cyber security aspects that go beyond generic IT aspects. Of note, the malicious sequence was processed by an NGS device (an Illumina NextSeq), but the sequencer itself was not used as a propagation vector nor was it compromised. Rather, it was the NGS device’s proper functionality that permitted the attack in the first place.

## Attack vectors

A schematic representation of the public health microbiological workflow appears in the **Figure**, involving sample preparation, sequencing and bioinformatics analysis stages [19]. The bioinformatics analysis usually involves an output or end result, which is interpreted and communicated to relevant stakeholders [20]. **Table 1** describes the different attack vectors and methods applicable to a generic NGS process. An adversary can attack at multiple stages of the NGS pipeline, with different attacks requiring different access levels (e.g. physical, local network, remote network). This analysis highlights the need for policymakers to employ cyber security best practices throughout the NGS diagnostic cycle, starting from the acquisition of biological material and ending in cloud-based bioinformatic applications. The analysis shown in **Table 1** is generic – different NGS platforms use a variety of technologies and architectures, making some of the threats relevant only to a subset of currently available platforms. All stages of the NGS process, from sample preparation to post-sequencing bioinformatics analysis, could be vulnerable to cyber attacks.

**Figure:**
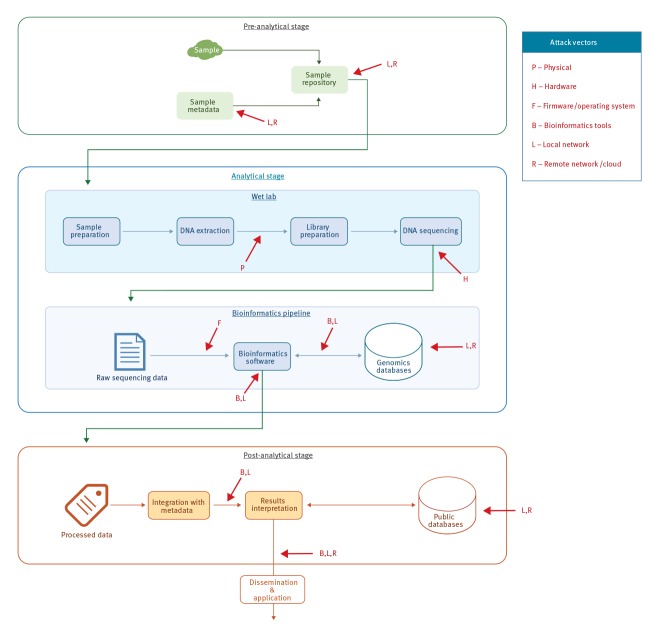
Cyber threat assessment in public health microbiology

Schematic representation of a sample-to-result microbiological workflow in the public health microbiology setting. The workflow is divided into the pre-analytical, analytical and post-analytical phases of the diagnostic cycle. Red arrows represent vulnerabilities in the different phases of the process to different cyber attack vectors.

**Table 1:** Cyber threat analysis relevant to next generation sequencing in public health

Attack vector	Methods	Target NGS stage	Required access
Physical	Malicious biological material	Sample preparation	Physical
Hardware	Hardware implant	Sequencing	Interdiction/manufacturing
Firmware/operating system	Firmware replacement	Sequencing Bioinformatics	Physical Interdiction/manufacturing Compromised PC
Software	Targeted infection Supply chain	Bioinformatics	Compromised PC Local Network Remote
Local network	Targeted infection Supply chain Data breach	Bioinformatics	Compromised PC Remote
Cloud infrastructure	Data breach	Bioinformatics	Remote

NGS: next generation sequencing; PC: personal computer.

**Table 2** presents a risk assessment for representative attack vectors at the different stages of the NGS process. The probability and impact of each attack are ranked on a scale of 1 to 5, each based on the expert opinion of the authors. High-probability scores were awarded to threats that require minimal access to carry out, have higher technological feasibility and for which stronger incentives exist among adversaries. High-impact scores were awarded to threats resulting in overall system compromise and particularly to those which made it possible to use the host PC as a cyber attack propagation vector and to threats with a wider national or international impact. Following the Common Vulnerability Scoring System (CVSS) 3.1 methodology [21], an overall score for each vector was obtained by multiplying its probability and impact scores. The different threats were then categorised into three groups according to the overall score, with scores ranging from 1 to 5 being considered minor threats, 6 to 15 representing moderately dangerous threats and scores of 16 to 25 representing major threats. A total of 12 threats have been included in the analysis, containing six main attack vectors comprising of several adversarial methodologies. Of these, three were deemed major, six moderate and another three minor threats. Attacks pertaining to peripheral or proprietary hardware present the most dangerous combination of required access, attack impact and probability and required resources, followed by attacks on sequencing software. Table 2 also includes a selection of factors that can mitigate the highlighted threats. Some factors, such as protecting PCs and cloud servers, are generic IT best practices, while some are specific to the NGS domain and its use of connected sequencing hardware.

**Table 2:** Probability and impact assessment of representative cyber attack vectors

Attack vector	Method	Possible impact	Impact scale	Required access	Mitigating factors	Impact	Probability	Score
Biological processing	Synthesis of malicious biomatter that would compromise device or sequencing software	From false results to full system compromise	Devices sequencing malicious biomatter	Access to biological samples to be sequenced by device	Chain of custody as biomatter is handled; software protections in sequencer	5	1	5
Signal processing	Flash malicious bitstream/hardware replacement	Misdetection of bases, false results	Single device	Physical access	Binding and tamper-proofing sequencer, signing and authenticating field upgrades	4	3	12
Proprietary hardware components	Flash malicious firmware on hardware subsystem	Misdetection of bases, false results	Single device	Access to a PC connected to the sequencer		4	3	12
	Feed sequencing software with false results	False-negative or false-positive result		Possibly accomplishable remotely	Authenticate device-PC communications	5	4	20
	Attack sequencing PC	Malicious code running on PC	Single device; possible propagation/ escalation vector		Standard practices for protecting PCs	5	5	25
Sequencing/bio-informatics software	Flash malicious firmware on subsystem	Misdetection of bases, false results	All devices in contact with malicious PC; possible propagation/ escalation vector	Access to a PC connected to the sequencer; possibly accomplishable remotely	Authenticate device-PC communications	5	3	15
	Display false sequencing results	False-negative or false-positive on detection of disease			Standard practices for protecting PCs	5	3	15
Sequencer and related equipment (e.g. PC)	Infect PC with targeted malware to interfere with sequencing software operations	False-negative or false-positive detection of disease; Ability to infect other devices and PCs	All devices and PCs on the same network as the malicious PC; network propagation/ escalation vector	Access to a PC connected to the sequencer; possibly accomplishable remotely	Restrict and regulate interface between PC and sequencer	5	2	10
	Propagate malware using sequencer as an infection vector	PCs in proximity of sequencer infected with malware	All PCs in contact with infected sequencer		Restrict and regulate interface between PC and sequencer	4	4	16
	Leak of sensitive personal data	Leak of sensitive personal data	Owner of sample/data		Standard practices for protecting PCs	2	5	10
	Report false data to the sequencer cloud	False data accumulated at scale, false global information	Commercial/public data repositories		Authenticate PC-cloud communications	2	1	2
Cloud services	Deliver malicious sequencer firmware or sequencing software at worldwide scale	Malicious software deployed at scale	All user base of a cloud, network propagation/escalation vector allows arbitrarily large infection scale	Remote	Standard practices for protecting cloud services	5	1	5

PC: personal computer.

Impact scale: 1 – minimal public health impact; 2 – local or limited consequences; 3 – moderate or severe local consequences; 4 – national consequences; 5 – severe national or international consequences.

Probability scale: 1 – minimal feasibility; 2 – limited feasibility and/or incentive; 3 – moderate feasibility and/or incentive; 4 – high feasibility and/or incentive; 5 – high feasibility, imminent.

## Attack objectives

The International Organization for Standardization (ISO) standards body defines in ISO/IEC 27000 a set of principles for the operation of a secure system: confidentiality, integrity and availability [8]. In the specific domain of NGS devices, several high-level motivations for an adversary can be considered according to these principles.

The **confidentiality** principle stipulates that a system must ensure that information is not made available or disclosed to unauthorised entities. In the context of NGS, attacks on confidentiality include data leakage of medical records, and especially of genetic information, which are considered to be highly personal and sensitive and thus of very high value. Data leakage may occur through the action of an outside attacker, but it may also occur through internal misuse (the ‘angry administrator’ scenario). Liabilities with respect to data safety and security are even more pronounced in light of the recent introduction of the general data protection regulations (GDPR). In the least harmful scenario, targeted advertising could take advantage of a person’s medical situation, maybe even without their awareness, to make profit. In a more concerning scenario, personal medical records of high-profile targets could be used to extort, blackmail or even physically harm them.

Beyond the individual level, leakage of raw sequence data or results of sequencing procedures, could result in an embarrassment to public health institutions, especially if information has not yet been properly analysed, or if information is presented out of context without relevant metadata and expert interpretation.

The **integrity** principle stipulates that a system must protect the accuracy and completeness of information. In the context of NGS, attacks on integrity include misdetection attacks, in which the device could appear to be functioning, while in effect, it provides false results to the user. Attacking a core sequencing facility intended for public health purposes, could lead to erroneous diagnosis and, as a consequence, mistreatment of patients or inconclusive investigation. Such a scenario would carry grave consequences both to individual patients and to medical and public health facilities. Significant economical and reputational damages should be taken into account in such situation.

Maintaining the integrity of devices is particularly important when they are used in an incident response scenario. As misdetection could result in a false alarm, e.g. an Ebola outbreak could be ‘detected’ while no actual virus was present, leading in an extreme case scenario to a public health response, disruption of routine and critical services, disruption of normal business, public panic and disorder and mobilisation of government resources to contain a non-existent outbreak. In an arguably worse-case scenario, misdetection may involve a false-negative result, meaning the sequencing procedure would report the sample as harmless, while it actually contained a significant biological threat.

The **availability** principle stipulates that a system should be accessible and usable when an authorised entity demands access. Denial of service is a form of attack in which a device, process, or facility is rendered unavailable. In our specific context, sequencing devices could be arranged to fail under certain conditions. At the very least, such an incident imposes an economic penalty on a victim organisation. Furthermore, an unexpected failure of devices during a biological incident can significantly delay or even deny appropriate public health response.

At the IT infrastructure scale, attackers may attempt to compromise a weakly secured device as a stepping stone for infiltrating a different network or system. In this scenario, the real objective of the attack will not be to attack the NGS device itself, but rather to achieve system or network compromise. In such an attack, the NGS device is used as an infection and propagation vector for advancing the attacker’s position to target a machine, facility or network associated with the device. This attack is common to all connected devices and is not unique to NGS devices. NGS devices, however, are mainly used in government and medical facilities, arguably two of the highest-risk sectors regarding cyber activity, making this threat important to consider. Moreover, the increasing popularity of mobile sequencers further augments this vulnerability.

It is also important to note that while attacks carried out on a single device would have a moderate impact at best, if deployed at scale, attacks may create a sustained incident on a national or even global level.

## Attack scenarios

Here we propose a number of possible attack scenarios and discuss the resources and skills required to carry them out.

### Biological substance attack

As demonstrated by Ney et al. [18], synthesising a malicious DNA sample to carry out an attack on a sequencing PC is technically feasible. That said, extensive knowledge of both computer science and microbiology is required to carry out such an attack, along with carrying out extensive security evaluation of the sequencing software to find a potential vulnerability. Furthermore, the malicious DNA sample should be tailored for the specific sequencing device on which the sample would end up, a non-trivial piece of foreknowledge. Finally, the question of how the sample would end up being synthesised by the device in the first place leads to scenarios involving field-deployed human agents or collaborators on the victim side. Those assumptions lead us to rate this threat as having a low probability of taking place. Nevertheless, the probability of such attack could increase in the future, depending on technological advancements.

### Malicious hardware/firmware implant

In this scenario, attackers manage to be in a position where they can communicate with the device locally, through serial or networked connections, or can physically disassemble it. Recent reports testify to the ability and motivation of state actors to place themselves in such positions [15,22]. It is not uncommon for workers of various sectors to use their company’s PCs for various personal activities, thus increasing the chance of infection by malware from the Internet: an NGS device compromised at time of manufacturing or by interdiction could serve as an infection vector for computing systems in a medical or government facility, but a PC infected ahead of time and controlled by the attacking party could be used as a remote implanting station for the NGS devices in its vicinity. In a typical public health laboratory setting, a small number of NGS devices will communicate with numerous PCs as part of sequencing and bioinformatics analysis stages, and so both directions are efficient propagation vectors. Most devices are typically protected from infection by IT security safeguards such as malware protection and secure coding practices. Medical devices, however, are known to be more sensitive to malware and low-quality code than other connected devices, owing to the lengthy compliance process that makes in-the-field upgrades very difficult [12]. Finally, embedded device firmware has been shown to suffer often from poor security mechanisms and thus is more susceptible to various forms of attacks than traditional computer systems [23]. The various factors described above lead us to believe that this attack scenario is highly probable.

### Next generation sequencing software compromise

Software is known to contain vulnerabilities caused by imperfect code, misconfiguration etc., and NGS-related software, used to operate sequencing and laboratory equipment or carry out the bioinformatics analyses, is no exception. Software vulnerabilities are exploited to gain unauthorised access to computer systems or networks, leak data, crash or otherwise disrupt various services. In the NGS context, vulnerable sequencing software could be made to malfunction, report false results or serve as an initial foothold on a medical or government facility’s network. If the application runs with high privileges or makes use of other high-privilege software components (e.g. a device driver), this scenario could lead to full system takeover. A remotely exploitable vulnerability could lead to a remote attacker controlling sequencing PCs across the world. At scale, this would mean any device which installed the sequencing application would serve as an entry point to its system and the network it attaches to. 

A different attack vector using the NGS software would be a supply chain attack similar to an incident reported in 2017 [24], in which the online software repository used to distribute a popular application was compromised, and the hosted application was replaced by a malicious version of itself. All instances of the application downloaded from the repository would infect their host PCs with malware. A similar incident can occur with the repository hosting software powering a benchtop or a portable sequencer. According to a recent audit of popular sequencing software packages performed by Ney et al. [18], those applications generally suffer from bad security hygiene practices and thus finding an exploit in one of them is highly feasible.

### Policy implications

The field of microbial genomics is vulnerable to cyber threats and therefore, there is a need to develop and implement a suitable policy to mitigate such threats. The main components of such policy may include the following:

- Cyber security aspects should be taken into account when local, national or international surveillance systems based on genomics are designed and implemented.
- NGS devices are not simple, passive devices – they contain active computing and networking capabilities and should thus be appropriately considered by IT policy. Good general IT and information security organisational practice is important to protect against many of the risks described herein.
- An ongoing dialogue between scientists and practitioners and IT and security personnel is needed in order to identify cyber threats related to newly developed and introduced technology.
- Skills and capacity building in cyber security should be considered by public health institutions and should be introduced to formal education programmes as well as on-the-job training.
- The possibility of a cyber attack should be taken into account during outbreak detection and investigation and explored further by specialists if deemed relevant.
- Manufacturers of laboratory equipment, particularly DNA sequencing technology, should consider cyber security threats during platform development, manufacturing and marketing.
- Developers of commercial or open source bioinformatics software should consider cyber security threats during software development and testing.
- Surveillance tools, capable of detecting or predicting cyber attacks involving DNA sequencing should be developed and implemented in surveillance networks.
- The impact and probability of the various attack vectors should be evaluated more broadly while consulting a range of experts from related fields in different countries, in order to fine-tune and validate risk assessments.

Given the rapid evolution of DNA sequencing technology and its applications for microbial genomics and in light of the dynamics of the cyber security domain, frequent risk assessments should be carried out in order to identify new threats and update public health policy aimed at mitigating those risks.

## References

[1] Chiu C, Miller S. Next-generation sequencing. In: Persing DH, Tenover FC, Hayden RT, Ieven M, Miller MB, Nolte FS, et al. (eds). Molecular microbiology: Diagnostic principles and practice. Washington: American Society of Microbiology. 2016. pp. 68-79.

[2] Motro Y Moran-Gilad J Next-generation sequencing applications in clinical bacteriology. Biomol Detect Quantif. 2017;14:1-6. 10.1016/j.bdq.2017.10.002 29255684 PMC5727008

[3] Moran-Gilad J Whole genome sequencing (WGS) for food-borne pathogen surveillance and control - taking the pulse. Euro Surveill. 2017;22(23):30547. 10.2807/1560-7917.ES.2017.22.23.30547 28661389 PMC5479979

[4] Xiong M Zhao Z Arnold J Yu F Next-generation sequencing. J Biomed Biotechnol. 2010;2010:370710. 10.1155/2010/370710 21512588 PMC3075819

[5] Moran-Gilad J How do advanced diagnostics support public health policy development? Euro Surveill. 2019;24(4):1900068. 10.2807/1560-7917.ES.2019.24.4.1900068 30696524 PMC6351996

[6] Woolf N. DDoS attack that disrupted internet was largest of its kind in history, experts say. London: The Guardian. 2016. Available from: https://www.theguardian.com/technology/2016/oct/26/ddos-attack-dyn-mirai-botnet

[7] Bambrough B Bitcoin and crypto wallets are now being targeted by malware. New Jersey: Forbes; 2019. Available from: https://www.forbes.com/sites/billybambrough/2019/09/19/bitcoin-and-crypto-wallets-now-targeted-by-malware/#697e906a65db

[8] International Organization for Standardization (ISO). ISO/IEC 27032:2012. Information technology – security techniques – guidelines for cybersecurity. Geneva: ISO; 2012. Available from: https://www.iso.org/standard/44375.html

[9] Jain M Olsen HE Paten B Akeson M The Oxford Nanopore MinION: delivery of nanopore sequencing to the genomics community. Genome Biol. 2016;17(1):239. 10.1186/s13059-016-1103-0 27887629 PMC5124260

[10] Castro-Wallace SL Chiu CY John KK Stahl SE Rubins KH McIntyre ABR Nanopore DNA sequencing and genome assembly on the International Space Station. Sci Rep. 2017;7(1):18022. 10.1038/s41598-017-18364-0 29269933 PMC5740133

[11] Coventry L Branley D Cybersecurity in healthcare: A narrative review of trends, threats and ways forward. Maturitas. 2018;113:48-52. 10.1016/j.maturitas.2018.04.008 29903648

[12] Martin G Martin P Hankin C Darzi A Kinross J Cybersecurity and healthcare: how safe are we? BMJ. 2017;358:j3179. 10.1136/bmj.j3179 28684400

[13] Field M. WannaCry cyber attack cost the NHS £92m as 19,000 appointments cancelled. London: The Telegraph; 2018. Available from: https://www.telegraph.co.uk/technology/2018/10/11/wannacry-cyber-attack-cost-nhs-92m-19000-appointments-cancelled/

[14] Markowsky G, Markowsky L. From air conditioner to data breach. In: Daimi K, Arabnia HR. (eds). Proceedings of the 2014 International Conference on Security and Management (SAM). Worldcomp'14; 21-24 Jul 2014; Las Vegas. Available from: http://docplayer.net/7778877-George-markowsky-ashu-m-g-solo-kevin-daimi-samiha-ayed-michael-r-grimaila-hanen-idoudi-editors-hamid-r-arabnia.html

[15] Appelbaum J, Gibson A, Guarnieri C, Müller-Maguhn A, Poitras L, Rosenbach M, et al. The Digital Arms Race. NSA preps America for future battle. Hamburg: Der Spiegel; 2015. Available from: https://www.spiegel.de/international/world/new-snowden-docs-indicate-scope-of-nsa-preparations-for-cyber-battle-a-1013409.html

[16] Finlayson SG Bowers JD Ito J Zittrain JL Beam AL Kohane IS Adversarial attacks on medical machine learning. Science. 2019;363(6433):1287-9. 10.1126/science.aaw4399 30898923 PMC7657648

[17] Mirsky Y, Mahler T, Shelef I, Elovici Y. CT-GAN: Malicious tampering of 3D medical imagery using deep learning. arXiv preprint arXiv:1901.03597;2019. Available from: https://arxiv.org/abs/1901.03597

[18] Ney P, Koscher K, Organick L, Ceze L, Kohno T. Computer security, privacy, and DNA sequencing: compromising computers with synthesized DNA, privacy leaks, and more. In: Proceedings of the 26th USENIX Security Symposium 16-18 Aug 2017; Vancouver. Available from: https://www.usenix.org/system/files/conference/usenixsecurity17/sec17-ney.pdf

[19] Knetsch CW, van der Veer EM, Henkel C, Taschner P. DNA sequencing. In: van Pelt-Verkuil E, van Leeuwen W, te Witt R. (eds). Molecular diagnostics. Singapore: Springer; 2019. pp. 339-360. Available from: https://link.springer.com/chapter/10.1007/978-981-13-1604-3_8

[20] Hadjadj L Baron SA Diene SM Rolain JM How to discover new antibiotic resistance genes? Expert Rev Mol Diagn. 2019;19(4):349-62. 10.1080/14737159.2019.1592678 30895843

[21] Forum of Incident Response and Security Teams (FIRST). Common Vulnerability Scoring System version 3.1: Specification Document. Cary: FIRST. [Accessed: 5 Feb 2020]. Available from: https://www.first.org/cvss/specification-document

[22] Robertson J Riley M The big hack: How China used a tiny chip to infiltrate US companies. Bloomberg Businessweek. 2018. Available from: https://www.bloomberg.com/news/features/2018-10-04/the-big-hack-how-china-used-a-tiny-chip-to-infiltrate-america-s-top-companies

[23] Shwartz O, Mathov Y, Bohadana M, Elovici Y, Oren Y. Opening Pandora’s box: effective techniques for reverse engineering IoT devices. In: Eisenbarth T, Teglia Y (eds). International Conference on Smart Card Research and Advanced Applications 13 Nov 2017; Montpellier. CARDIS 2017. Lecture Notes in Computer Science, vol 10728. Cham: Springer. Available from: https://link.springer.com/chapter/10.1007/978-3-319-75208-2_1#citeas

[24] Brumaghin E, Gibb R, Mercer W, Molyett M, Williams C. CCleanup: A vast number of machines at risk. San Jose: Cisco TALOS. 2017. Available from: https://blogs.cisco.com/security/talos/ccleanup-a-vast-number-of-machines-at-risk

